# Comparison of outcome between blood culture positive and negative infective endocarditis patients undergoing cardiac surgery

**DOI:** 10.1186/s13019-021-01532-9

**Published:** 2021-05-27

**Authors:** Kristians Meidrops, Arina Zuravlova, Janis Davis Osipovs, Martins Kalejs, Valerija Groma, Eva Petrosina, Aigars Reinis, Eva Strike, Uga Dumpis, Andrejs Erglis, Peteris Stradins

**Affiliations:** 1 Riga Stradins University, 16 Dzirciema Street, Riga, LV-1007 Latvia; 2 Centre of Cardiac Surgery, Pauls Stradins Clinical University Hospital, 13 Pilsonu Street, Riga, LV-1002 Latvia; 3 Statistics Unit, Riga Stradins University, 14 Balozu Street, Riga, LV-1007 Latvia; 4 Faculty of Physics, Mathematics and Optometry, UL House of Science, University of Latvia, 3 Jelgavas Street, Riga, LV-1004 Latvia; 5 Department of Biology and Microbiology, Riga Stradins University, 16 Dzirciema Street, Riga, LV-1007 Latvia; 6 Department of Cardiovascular Anaesthesia and Intensive Care, Pauls Stradins Clinical University Hospital, Riga, LV-1002 Latvia; 7 Department of Infection Control, Pauls Stradins Clinical University Hospital, 13 Pilsonu Street, Riga, LV-1002 Latvia; 8 Centre of Cardiology, Pauls Stradins Clinical University Hospital, 13 Pilsonu Street, Riga, LV-1002 Latvia; 9 Faculty of Medicine, UL House of Science, University of Latvia, 3 Jelgavas Street, Riga, LV-1004 Latvia

**Keywords:** Infective endocarditis, Blood culture negative, Blood culture positive, Procalcitonin, Intrahospital mortality, Long-term mortality

## Abstract

**Background:**

Up to 30% or even more of all infective endocarditis (IE) cases are recognized as blood culture negative, meaning that the causative agent is left unidentified. The prompt diagnosis together with the identification of causative microorganism and targeted antibiotic treatment can significantly impact the prognosis of the disease and further patient’s health status. In some studies, blood culture negative endocarditis has been shown to be associated with delayed diagnosis, worse outcome and course of the disease, and a greater number of intra and postoperative complications.

**Methods:**

We retrospectively analysed the medical records of all patients who underwent cardiac surgery for endocarditis between years 2016 and 2019. The aim of this study was to analyse short and long-term mortality and differences of laboratory, clinical and echocardiography parameters in patients with blood culture positive endocarditis (BCPE) and blood culture negative endocarditis (BCNE) and its possible impact on the clinical outcome.

**Results:**

In our study population were 114 (55.1%) blood culture positive and 93 (44.9%) blood culture negative cases of infectious endocarditis. The most common pathogens in the blood culture positive IE group were *S.aureus* in 36 cases (31.6%), *Streptococcus spp*. in 27 (23.7%), *E.faecalis* in 24 (21.1%), and other microorganisms in 27 (23.7%). Embolic events were seen in 60 patients (28.9%). In univariate analyses, detection of microorganism, elevated levels of procalcitonin were found to be significantly associated with intrahospital death, however it did not reach statistical significance in multivariate analyses. Among microorganisms, *S.aureus* was significantly associated with intrahospital death in both univariate and multivariate analyses.

**Conclusions:**

There are no statistically significant differences between groups of BCPE and BCNE in terms of intrahospital mortality, hospital and ICU stay or 3-year mortality.

There were higher levels of procalcitonin in BCPE group, however procalcitonin failed to show independent association with mortality in multivariate analysis.

The most common microorganism in the BCPE group was *S.aureus.* It was associated with independently higher intrahospital mortality when compared to other causative microorganisms.

## Background

Infective endocarditis (IE) is an infection of the heart valves and inner layer of heart chambers. Even though IE can be caused by any type of bacteria or fungi, the majority of cases are caused by a small number of bacterial species. The most common bacterial species are *S. aureus*, followed by viridans group streptococci and enterococci [1]. Pathogenic bacteria and fungi attach to previously formed lesions and together with platelets and fibrin form vegetations, cause valvular dysfunction, which can manifest as heart failure. Subsequently under the impact of turbulent blood flow, pressure and vegetation instability a rupture may occur in vegetations thus forming emboli. Those in turn may cause septic emboli and ischemic areas in various organ systems [2, 3].

In developed countries, the incidence of IE ranges from 4 to 7 cases per 100,000 population per year and has remained stable during recent decades. A relatively rare disease, it still leaves a high impact on an affected patient’s life with high mortality rate up to 9–30% [2, 4].

Modified Duke criteria nowadays are being used to diagnose IE. Microbiological detection of a causative agent (MDCA) and positive imaging results are the main criteria. Establishing a diagnosis of definite IE is challenging for physicians as MDCA is not always possible to fulfil.

Up to 30% or more of IE cases might be blood culture negative. It can be due to low bacteraemia at the moment of blood draw or due to previously initiated antibacterial treatment, fastidious, intracellular pathogens or because the type of medium is not appropriate for some rarer forms of bacteria. It is reported that IE could be even polymicrobial in up to 5% of cases [2, 5–7].

Some studies propose microorganism detection algorithms, which seek to increase microorganism identification level thereby decreasing BCNE rate. It should be considered whether microorganism detection by these specific algorithms is clinically relevant and cost effective in cases where diagnosis of IE can be made based on ordinary imaging and clinical findings. If any additional detection methods are being used, it is important to determine whether possible results will significantly change a treatment plan. Patient risk factor identification and atypical causative microorganism detection could be beneficial for developing a more precise and effective treatment strategy depending on the clinical situation [8].

The prompt diagnosis together with identification of IE causative agent and targeted antimicrobial treatment can significantly impact the prognosis of the disease and further on patient’s health status. In some studies, blood culture negative endocarditis is associated with delayed diagnosis, worse outcome and course of the disease, greater number of intra- and postoperative complications [8, 9].

On the other hand, there are studies which suggest that BCNE and BCPE have no difference in short and long-term survival. These papers note that other independent factors such as diabetes mellitus contribute more to the clinical course than microorganism detection alone [10, 11].

Other authors also point out certain laboratory findings that could be more commonly seen in BCNE such as lower CRP levels and higher BNP levels [12, 13].

Therefore, the aim of this study was to analyse short and long-term mortality and differences of laboratory, clinical and echocardiography parameters in patients with BCPE and BCNE and its possible impact on the clinical outcome.

## Methods

We conducted a retrospective analysis of medical records of all 207 IE patients who underwent cardiac surgery at Pauls Stradins Clinical University Hospital, Latvia, between years 2016 and 2019. Our centre is the only one providing adult cardiac surgery in Latvia thus representing the whole surgically treated infective endocarditis patient population in Latvia. Two groups of patients were defined – patients with BCPE and patients with BCNE. In our hospital no Endocarditis team was available during the time of the study. Diagnosis of IE was made by cardiologists and cardiac surgeons and specialists from different specialities depending on patients’ clinical status and comorbidities. Definite and possible IE diagnosis was based on modified Duke criteria.

All the data from laboratory analyses refer to the last samples taken prior surgery. The study was approved by the Ethical Committee of the Hospital (Decision No. 230419-17 L).

### Statistical analysis

Categorical variables were expressed as relative frequencies (percentages), for quantitative variables mean (standard deviation [SD]) and median (interquartile range [IQR]) were used for description of normally and non-normally distributed data, respectively. Graphical tools were used for assessing normality. For categorical variables accordingly to tests assumptions Chi-square test or Fisher exact test was used to compare difference between groups. For quantitative variables accordingly to test assumptions t-test or Mann-Whitney U test was used to compare groups. Univariate and multivariate logistic regression was used to see association between potential factors that might affect survival (intrahospital death). *P*-values below 0.05 were considered statistically significant. Statistical analyses were performed using IBM SPSS version 26. RStudio, version 1.3.1073 was used to depict the results of survival analysis.

## Results

We collected medical records of 207 patients who underwent cardiac surgery for IE. There were 114 (55.1%) blood culture positive and 93 (44.9%) blood culture negative cases of infectious endocarditis. The rate of pathogen detection from tissue cultures obtained at the time of surgery was low. In the BCNE group pathogen from tissue culture was detected in 5.4 and 13.2% in the BCPE group.

Preoperatively all BCPE cases and 71 of 93 (76.3%) BCNE cases were classified as definite infective endocarditis. Twenty-two cases of BCNE were classified as possible infective endocarditis and met definitive endocarditis criteria when confirmed histologically.

Necessity for cardiac surgery was based on three main indications suggested in 2015 ESC European guidelines for the management of infective endocarditis – heart failure, uncontrolled infection and prevention of embolism. As shown in Table 1, indications for cardiac surgery most often were combined, leaving isolated indication such as heart failure due to valvular dysfunction and isolated prevention of embolism only in the minority of cases of left heart side IE.

**Table 1:** Indications for cardiac surgery in patients with IE

Heart failure due to valvular dysfunction	5.80%
Prevention of embolism	3.86%
Heart failure due to valvular dysfunction and prevention of embolism	41.06%
Heart failure due to valvular dysfunction and prevention of embolism and uncontrolled infection	28.02%
Heart failure due to valvular dysfunction and uncontrolled infection	20.29%
Uncontrolled infection and prevention of embolism	0.97%

Table 2 summarises the patients’ characteristics. In the BCPE group there were significantly higher number of patients with intravenous drug addiction as well as a prevalence of HCV infection. Among comorbidities type 1 diabetes mellitus rate was significantly higher in BCPE group patients (Table 3). The most commonly detected pathogens were *S.aureus* in 36 (31%) cases, *Streptococcus spp.* in 27 (24%), *E.faecalis* in 24 (21%) and other microorganisms in 27 (24%) cases. There was no statistically significant difference between groups of BCPE and BCNE regarding locally uncontrolled infection, vegetation size, embolic events and haemodynamic instability (Table 4). Even though patients with embolic events had bigger vegetation size, we did not find significant association. Mean vegetation size among patients who had embolic events was 16.0 (11.3) mm versus 15.5 (7.9) mm among those who did not (*p* = 0.795). Altogether embolic events were seen in 60 patients (28.9%). In laboratory analyses, significantly higher levels of procalcitonin and lower levels of haemoglobin and haematocrit were observed in the BCPE group (Table 5). Intrahospital mortality in the BCPE group was 14.04% in comparison with 5.38% in the BCNE group, however it did not reach statistical significance (*p* = 0.062) (Table 6).

**Table 2:** Characteristics of IE patients depending on blood culture status

	Blood culture positive	Blood culture negative	*p* value
Age, mean (years)	57.17 (15.59)	53.61 (12.80)	0.073
Sex, male, %	78.07	65.59	**0.046**
BMI, mean (kg/m^2^)	25.45 (4.48)	25.91 (5.65)	0.531
PVE, %	21.51	19.30	0.694
Aortic valve IE, %	38.60	41.94	0.626
Mitral valve IE, %	32.46	22.58	0.116
Aortic and mitral valve IE, %	18.42	29.03	0.072
Left heart side IE, %	89.47	95.70	0.095
Intravenous drug injection history, %	12.28	4.30	**0.043**
Haemodynamically stable, %	88.60	92.47	0.348
Left ventricle ejection fraction, mean, %	55.24 (11.19)	55.77 (9.18)	0.714
Euroscore II risk, mean, %	7.35	6.80	0.595

**Table 3:** Comorbidities in BCPE and BCNE patients

	Blood culture positive	Blood culture negative	*p* value
Diabetes mellitus, type 1, %	5.56	0.00	**0.032**
Diabetes mellitus, type 2, %	9.26	8.79	0.909
HIV infection, %	6.42	1.10	0.074
HCV infection, %	15.74	5.49	**0.022**
HBV infection, %	0.00	1.10	0.457
Spondylodiscitis, %	5.26	1.08	0.132
Oncology, %	4.39	3.23	0.733
Arterial hypertension, %	4.59	12.09	0.720

**Table 4:** Occurrence of embolic events, locally uncontrolled infection, haemodynamic instability prior surgery and vegetation size in IE patients depending on blood culture status

	Blood culture positive	Blood culture negative	*p* value
Embolic events prior to surgery, %	29.82	27.96	0.768
Locally uncontrolled infection, %	30.00	31.87	0.775
Haemodynamically unstable, %	11.40	7.53	0.348
Vegetation size, mm, mean	16.70 (9.95)	14.48 (7.69)	0.097

**Table 5:** Laboratory analyses in IE patients depending on blood culture status

	Blood culture positive	Blood culture negative	*p* value
CRP, mg/L	37.90 (9.98–94.33)	25.00 (12.25–63.70)	0.206
Procalcitonin, ng/ml	0.45 (0.10–2.33)	0.10 (0.10–0.50)	**0.001**
BNP, pg/ml	560.45 (157.73–1580.05)	469.50 (178.35–1114.30)	0.424
Creatinine, μmol/L	85.50 (63.25–107.75)	87.50 (66.00–110.00)	0.734
Glucose, mmol/L	6.08 (1.70)	6.20 (1.56)	0.614
Bilirubin, μmol/L	8.00 (6.00–15.00)	8.00 (6.00–12.00)	0.592
Leukocyte count × 10^9/L	9.20 (4.15)	8.81 (3.73)	0.484
Haemoglobin, g/L	103.85 (23.00)	110.91 (21.09)	**0.024**
Haematocrit, %	32.12 (6.07)	34.06 (6.41)	**0.029**
Platelet count × 10^9/L	261.73 (129.83)	256. 58 (110.58)	0.763

Mean (SD) or Med (IQR) used

**Table 6:** Duration of hospital and ICU stay, reoperation for bleeding and mortality rates in IE patients depending on blood culture status

	Blood culture positive	Blood culture negative	*p* value
Intrahospital stay, days, mean	25.00 (16.00–35.00)	23.00 (17.50–31.00)	0.378
Stay in ICU, days, mean	2.00 (1.00–5.00)	2.00 (1.00–3.00)	0.828
Reoperation for bleeding, %	17.54	12.90	0.358
Mortality, %	14.04	5.38	0.062

**Fig. 1:**
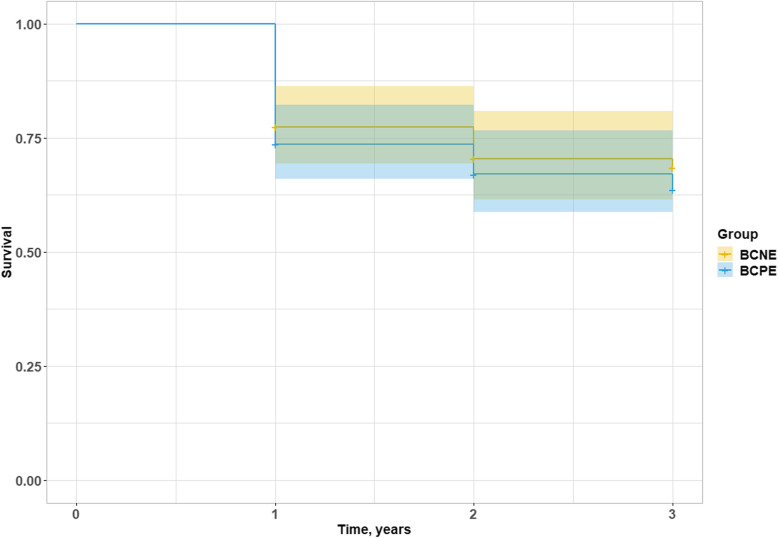
Long-term survival in surgically treated BCPE and BCNE patients

Microorganism detection and higher procalcitonin level in univariate analysis showed an association with intrahospital death, however it did not feature as an independent predictor of mortality in multivariate analysis (Table 7). Among microorganisms, *S.aureus* was independently associated with intrahospital mortality (Table 8). In long-term period (Fig. 1) better survival was seen in BCNE, however, statistical significance was not found (*p* = 0.509).

**Table 7:** Predictors of hospital mortality by univariable and multivariable logistic regression analyses

Variable	Univariable OR (95% CI)	*p* value	Multivariable adjusted OR (95% CI)	*p* value
Microorganism detection	2.873 (1.011–8.167)	**0.048**	1.166 (0.319–4.272)	0.816
Age	1.024 (0.991–8.167)	0.155	1.027 (0.975–1.082)	0.319
Body mass index	1.034 (0.946–1.129)	0.466	1.034 (0.912–1.173)	0.599
CRP (C-reactive protein)	1.003 (0.995–1.011)	0.490	1.002 (0.991–1.014)	0.696
Procalcitonin	1.036 (1.004–1.068)	**0.025**	1.033 (0.999–1.068)	0.057
Leukocytes	1.029 (0.924–1.146)	0.608	1.066 (0.909–1.249)	0.433
Embolism	1.586 (0.621–4.048)	0.335	1.311 (0.368–4.673)	0.677
Intravenous drug user	0.497 (0.063–3.937)	0.508	1.202 (0.087–16.600)	0.891
Prosthetic valve endocarditis	2.157 (0.810–5.742)	0.124	0.849 (0.024–29.994)	0.928
Native valve endocarditis	0.464 (0.174–1.234)	0.124	0.840 (0.025–27.790)	0.922

**Table 8:** Detected microorganisms as predictors of hospital mortality by univariable and multivariable logistic regression analyses

Variable	Univariable OR (95% CI)	*p* value	Multivariable adjusted OR (95% CI)	*p* value
*E.faecalis*	2.325 (0.527–10.246)	0.265	1.310 (0.356–4.824)	0.685
*Streptococcus spp.*	1.788 (0.422–7.587)	0.430	1.073 (0.295–3.910)	0.915
*S.aureus*	4.408 (1.406–13.821)	**0.011**	3.332 (1.268–8.751)	**0.015**
Other microorganism	1.302 (0.243–6.962)	0.758	0.678 (0.149–3.089)	0.615

## Discussion

In this retrospective study, we analysed BCPE and BCNE patients’ data and clinical outcomes undergoing cardiac surgery. Of 207 patients, 93 (44.9%) were BCNE. In literature, the rate of BCNE is described as greatly varying from 2.5 up to 70%, indicating a high rate of BCNE in this study [14].

In the study of Fournier et al. the rate of BCNE within a prospective analysis of 918 patients was 30.8%. Their proposed diagnostic strategy in 283 patients with BCNE found aetiology in 138 patients (78.0%) in whom 135 had causative microorganism identified. Of these, intracellular bacteria as *C.burnetii*, *Bartonella spp*, and *T.whipplei* accounted for 15.9% cases of BCNE. In 3 patients (1.1%), the aforementioned authors confirmed non-infectious aetiology of endocarditis. In BCNE, 56 of 70 patients infected with gram-positive cocci had received antibiotics prior to blood culture collection, indicating early antibiotics administration as a possible cause of BCNE [8]. During the time period of the study we used conventional methods of bacteriological examination of culturable pathogens without any extended diagnostic strategy, which partly explains the high incidence of BCNE and low detection of intracellular bacteria. Data published by Lamas et al. suggested that BCNE resulted mostly from priorly administered antibiotics and had an association with severe haemodynamic compromise [11]. Since many patients were transferred to our hospital from different institutions, we failed to assess priorly administered antibiotics from medical records. This could play a major role in our study, resulting in lower inflammatory markers and less severe preoperative condition in BCNE group.

Data in literature for BCNE patients regarding the outcome are controversial. Our study findings indicate no statistically significant difference of intrahospital mortality and long-term mortality between BCPE and BCNE patients. However, there was a tendency towards a worse outcome in the BCPE group. In univariate analysis there were higher rates of intrahospital mortality, although it failed to show statistical significance in multivariate analysis. This finding can possibly be explained by a higher severity of preoperative condition and less controlled infection in the BCPE group. We observed significantly lower haemoglobin and haematocrit levels prior to surgery, a higher procalcitonin level (Table 5) and other factors such as drug addiction (Table 2) and the presence of comorbidities (Table 3). Whilst not statistically significant, there were higher rates of haemodynamic instability and higher CRP levels in the current study. Our findings are in line with the results observed by Phua et al. characterizing outcomes of culture negative versus culture positive sepsis [15].

Higher inflammatory markers such as CRP and procalcitonin are usually associated with more severe preoperative conditions. The usefulness of a procalcitonin marker has been explored by other authors when studying IE and sepsis [16–18]. Recently, procalcitonin has been recognized as the most studied and suitable biomarker regarding antibiotic stewardship [19, 20]. There are studies suggesting that higher baseline CRP levels are independently associated with short term adverse events and higher procalcitonin levels are independently associated with increased intrahospital mortality [12, 21]. R.F.Siciliano et al., in a prospective study of 221 episodes, BCNE and BCPE were compared and a BCNE rate of 23.1% was reported. They have found lower CRP levels at the time of admission in BCNE group, which is consistent with the results of our study [13]. Our study showed an increased procalcitonin level in association with mortality in the univariate analysis, however it failed to show it in the multivariate analysis (Table 7).

The most commonly detected microorganism was *S.aureus* (31.6%), which was significantly associated with intrahospital death among other microorganisms in both univariate and multivariate analyses (Table 8). This finding is in agreement with the results demonstrated by other authors [22]. *S.aureus* is a pathogen known for its aggressive clinical presentation and worse prognosis compared to other microorganisms [23]. A high proportion of aggressive microorganism such as *S.aureus* in BCPE group could impact outcome results and might be associated with more severe preoperative conditions. Multi-antibiotic resistance in our study did not seem to impact the outcome, since there were only few multiresistant microorganism caused IE cases.

Two major limitations of this study are the relatively small sample size and a single center study. There are however very few studies published regarding this topic, especially studying the role of BCNE among the patients’ undergoing cardiac surgery. Our cardiac surgery center is the only one providing adult cardiac surgery services in the country and is therefore representing the whole surgically treated infective endocarditis patient population in Latvia.

## Conclusions

There are no statistically significant differences between groups of BCPE and BCNE in terms of intrahospital mortality, hospital and ICU stay or 3-year mortality.

Although BCPE patients have higher intrahospital and long-term mortality than BCNE patients, BCPE is not independently associated with mortality in multivariate analysis.

There were higher levels of procalcitonin in BCPE group, however procalcitonin failed to show independent association with mortality in multivariate analysis.

The most common microorganism in the BCPE group was *S.aureus.* It was associated with independently higher intrahospital mortality (OR rate of 3.332 and 4.408 in uni- and multivariate analyses) when compared to other causative microorganisms.

## Data Availability

The dataset used and analysed during the current study is available from the corresponding author upon reasonable request.

## Abbreviations

IE: Infective endocarditis
BCPE: Blood culture positive infective endocarditis
BCNE: Blood culture negative infective endocarditis
NVE: Native valve endocarditis
PVE: Prosthetic valve endocarditis
CRP: C-reactive protein
MDCA: Microbial detection of causative agent
SD: Standard deviation
IQR: Interquartile range
HIV: Human immunodeficiency virus
HCV: Hepatitis C virus
HBV: Hepatitis B virus
BMI: Body mass index
ICU: Intensive care unit
BNP: B-type natriuretic peptide
